# Apoptosis Modulation by LL-37: From Mitochondrial Cell Death to Prosurvival Signaling

**DOI:** 10.3390/ijms27188351

**Published:** 2026-09-19

**Authors:** Olga Evgenevna Voronko, Vitalia Sergeevna Novikova, Olesya Olegovna Klychkova, Margarita Pavlovna Markina, Arthur Anatolievich Lee, Dmitry Alexandrovich Kashirskikh, Victoria Alexandrovna Khotina, Vagif Ali oglu Gasanov

**Affiliations:** Koltzov Institute of Developmental Biology of the Russian Academy of Sciences, Moscow 119334, Russia; taqpolimeraza@gmail.com (O.E.V.); reddkk@bk.ru (V.S.N.); olesya_k8@mail.ru (O.O.K.); mmarkina46@gmail.com (M.P.M.); aal1999arth@gmail.com (A.A.L.); dim.kashirsckih@gmail.com (D.A.K.); gasanovvagif@gmail.com (V.A.o.G.)

**Keywords:** antimicrobial peptides (AMP), human cathelicidin, LL-37, apoptosis, cell death

## Abstract

This review summarizes current knowledge on the role of human cathelicidin LL-37 in the regulation of apoptosis. LL-37 can modulate programmed cell death pathways, exerting either proapoptotic or antiapoptotic effects depending on various factors, such as cell type and peptide concentration. Data on LL-37 derivatives and their involvement in apoptosis are also discussed. Understanding the molecular mechanisms underlying LL-37 regulation of apoptosis is necessary to expand the therapeutic potential of this peptide in various pathological conditions, including cancer.

## 1. Introduction

Apoptosis is a tightly regulated, non-inflammatory mechanism of programmed cell death in response to developmental or pathological signals. This process is triggered by irreparable cellular damage. Apoptosis plays an important role in regulating various physiological and pathological processes, including the immune response, prevention of uncontrolled cell proliferation, tissue homeostasis, and the elimination of damaged or infected cells [1]. Currently, there is an active search for ways to target apoptotic pathways to treat diseases such as cancer, neurodegenerative disorders, and autoimmune conditions. Elucidating the mechanisms by which apoptosis-related molecules and pathways could be modulated opens up opportunities for the development of innovative treatments and paves the way for targeted therapies, offering hope for curing diseases in which apoptosis is involved.

Cathelicidins are cationic cellular defense proteins that play a key role in innate host defense while also being associated with the pathogenesis of certain chronic diseases. These antimicrobial peptides (AMPs) possess microbicidal potential and the ability to modulate innate immunity and inflammatory processes. LL-37, the only human cathelicidin, is a peptide with a broad spectrum of antimicrobial activity, which also exerts immunomodulatory effects on cells and participates in inflammatory and anti-inflammatory processes, wound healing, and the induction of angiogenesis [2,3].

Beyond its bactericidal activity, LL-37 can bind to zwitterionic membranes of eukaryotic cells and exert cytotoxic effects on human peripheral leukocytes and MOLT T cells [4], as well as cause lysis of human erythrocytes [5]. Human cathelicidin has previously been shown to modulate cell death pathways [6,7]. LL-37 can induce apoptosis in various cells through different mechanisms depending on their type. The apoptotic effect of LL-37 may depend on factors such as the presence of bacteria, specific cell types, and peptide concentration. The proapoptotic effect of LL-37 has previously been demonstrated in vascular smooth muscle cells, periodontal ligament cells, neutrophils, T cells, and airway epithelial cells [7,8,9,10,11]. In contrast, several studies have shown an antiapoptotic effect of LL-37 [12,13,14].

The signaling pathways and mechanisms involved in LL-37-mediated apoptosis are not yet fully understood. Gaining insight into these processes would expand our knowledge of the role of LL-37 in various pathological conditions, including cancer. This review aims to provide a concise overview of the molecular aspects of the involvement of human cathelicidin LL-37 in apoptotic mechanisms.

## 2. Interaction of LL-37 with the Cell Membrane and Entry into the Cell

The dual ability of LL-37 to both induce and suppress apoptosis is largely determined by how the peptide interacts with the cell—whether it remains on the surface and activates receptors or enters the cell and acts on intracellular targets. Depending on its concentration and the lipid composition of the membrane, LL-37 can bind to plasma membrane receptors or insert into the bilayer to form pores, or penetrate into the cytosol via endocytosis.

One of the key factors determining the selective toxicity of LL-37 toward cells is the difference in lipid membrane composition and its net surface charge. The membranes of healthy eukaryotic cells normally have a zwitterionic character, whereas the membranes of many cancer cells are negatively charged due to the overexpression of anionic molecules such as phosphatidylserine, sialic acids, and sulfated glycosaminoglycans [15,16]. In the slightly acidic microenvironment characteristic of many solid tumors, the histidine residues of LL-37 become additionally protonated, enhancing its positive charge and affinity for tumor membranes [17]. In contrast, binding of the amphiphilic peptide to neutral or zwitterionic membranes of healthy cells is significantly weaker, which may contribute to preferential LL-37 binding to membranes of malignant cells.

LL-37 can interact with several receptors on the plasma membrane, including FPR2, P2X7, CXCR4, Toll-like receptors TLR3 and TLR4, MrgX2, and EGFR [18,19,20,21,22,23]. At low concentrations (0.01–5 μg/mL), the peptide remains on the surface, causing allosteric changes in receptors, promoting their translocation into lipid rafts, or affecting the mobility of receptor complex components, thereby triggering survival signaling pathways. Changes in plasma membrane fluidity upon LL-37 exposure can activate various downstream signaling cascades. Some enzymes are sensitive to alterations in lipid packing within the plasma membrane in the presence of membrane-active peptides [24]. At higher concentrations (≥10 μg/mL), LL-37 increases plasma membrane permeability. This is thought to occur either through oligomerization and the formation of transmembrane pores (the barrel-stave or toroidal pore models) or via the carpet model, in which the peptide accumulates on the membrane surface and, upon reaching a critical concentration, causes membrane destabilization in a detergent-like manner [25].

The mode of AMP–membrane interaction is determined by the lipid composition of the membrane: pore formation in unsaturated or cholesterol-containing lipids or membrane modulation in saturated lipids, leading to the formation of peptide–lipid tubes and fibrillar superstructures. Mixed aggregation (fiber formation) of LL-37 with lipids appears to be the preferred mode of action [26,27].

Beyond pore formation and membrane destabilization, LL-37 has recently been shown to disrupt epithelial integrity through a distinct mechanism. Kilari et al. (2025) [28] demonstrated that LL-37 triggers endocytosis and lysosomal degradation of tight junction proteins (such as claudin-1 and occludin) in colonic epithelial cells, leading to increased paracellular permeability. This process occurs independently of classical apoptosis or necrosis but may predispose cells to subsequent cell death pathways. Thus, LL-37 can compromise cell viability not only through direct membrane permeabilization but also by dismantling intercellular junctions.

LL-37 is imported into the cell via endocytosis mediated by both clathrin and caveolae [29,30]. Peptide endocytosis can occur at lipid rafts [29,31,32,33], with sulfated proteoglycans likely playing a key role in guiding peptide attachment and uptake [33]. In human macrophages, endocytosis of LL-37 appears to be partially dependent on the P2X7 receptor [29]. Another pathway involves endocytosis of the peptide together with the membrane receptor CXCR2. LL-37 endocytosis has been shown to occur via clathrin- and caveolin-dependent pathways involving lipid rafts with the formation of early Rab5+ endosomes expressing APPL1 and EEA1. In a study involving the induction of monocyte differentiation into monoosteophils, LL-37 underwent endocytosis into monocytes over 6 days, forming large (10 × 2 μm) specialized vesicles expressing LL-37 and integrin α3 [34].

Once in the cytosol, LL-37 can directly interact with mitochondrial membranes and target proteins, or can translocate to the nucleus, where it binds to gene promoter regions and G-quadruplexes [35,36].

## 3. Apoptosis and Proapoptotic Action of LL-37

**Figure 1:**
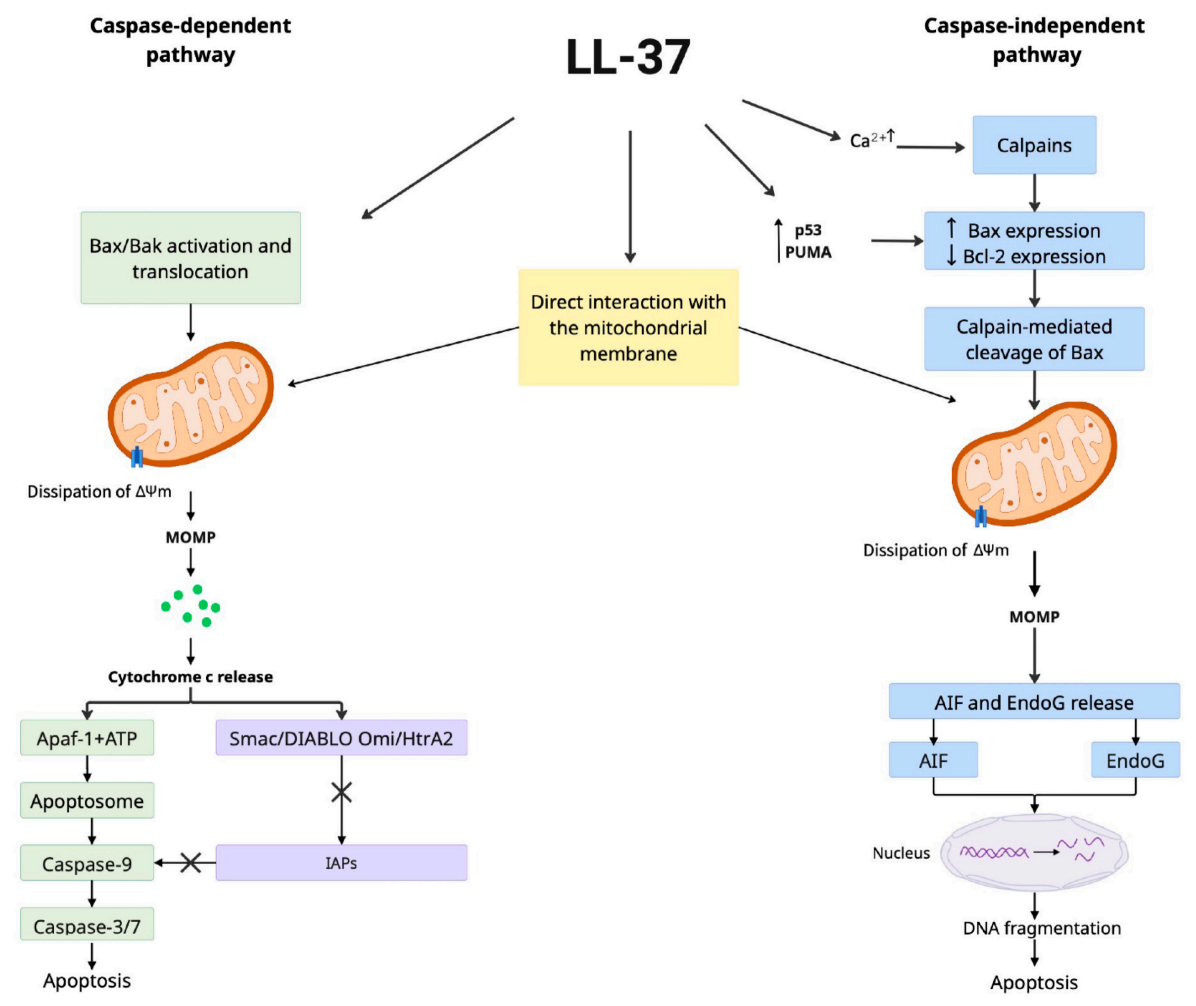
Mitochondrial pathways of LL-37-induced apoptosis. LL-37 can promote mitochondrial outer membrane permeabilization through Bax/Bak activation and mitochondrial translocation, direct interaction with the mitochondrial membrane, and Ca^2+^/calpain- and p53/PUMA-dependent signaling. In the caspase-dependent pathway, mitochondrial outer membrane permeabilization and loss of the mitochondrial membrane potential lead to the release of cytochrome c, Smac/DIABLO, and Omi/HtrA2. Cytochrome c, together with APAF-1 and ATP, promotes apoptosome formation and subsequent activation of caspase-9 and effector caspases-3 and -7. Smac/DIABLO and Omi/HtrA2 facilitate caspase activation by inhibiting inhibitor of apoptosis proteins. In the caspase-independent pathway, mitochondrial permeabilization results in the release of apoptosis-inducing factor and endonuclease G, followed by their translocation to the nucleus, DNA fragmentation, and apoptosis. Abbreviations: AIF, apoptosis-inducing factor; APAF-1, apoptotic protease-activating factor 1; EndoG, endonuclease G; IAPs, inhibitor of apoptosis proteins; MOMP, mitochondrial outer membrane permeabilization; ΔΨm, mitochondrial membrane potential. Symbols: arrows indicate the direction of signaling, translocation, release, or downstream events; crossed connectors (×) indicate inhibitory interactions; ↑ and ↓ indicate increased and decreased expression or levels, respectively.

Two well-characterized pathways of apoptosis are known: (1) the extrinsic (death receptor-mediated) pathway and (2) the intrinsic (mitochondria-mediated) pathway [1]. The major caspase-dependent and caspase-independent mechanisms of the intrinsic mitochondrial pathway are summarized in Figure 1.

The extrinsic pathway is mediated by caspase activation via receptors such as Fas or TNFR; however, to date, there are no published data on any interaction of LL-37 with these receptors. In contrast, the proapoptotic effect of LL-37 is primarily exerted through the intrinsic pathway, which will be discussed in detail below.

The mitochondrial pathway is associated with disruption of the integrity of the outer mitochondrial membrane and release of proapoptotic factors, leading either to activation of the caspase cascade in the cytosol (caspase-dependent pathway) or to the release and nuclear translocation of AIF and EndoG followed by DNA fragmentation, initiation of proapoptotic protein gene expression and subsequent involvement of these proteins in the execution of the apoptotic program (caspase-independent pathway) [1,37].

### 3.1. Mitochondrial Caspase-Dependent Apoptotic Pathway

#### 3.1.1. General Mechanism of Caspase-Dependent Apoptosis

Stress signals (ROS, DNA damage, ER stress, growth factor deprivation, etc.) lead to the accumulation of proapoptotic Bcl-2 family proteins, such as Bax and Bak, and their translocation from the cytosol to the mitochondria [1,6,38]. Bax/Bak are effector molecules that can change conformation, oligomerize, and form lipid pores in the outer mitochondrial membrane, which causes mitochondrial outer membrane permeabilization (MOMP) and leads to dissipation of the mitochondrial transmembrane potential (ΔΨm) [38,39].

A key event in the mitochondrial mechanism of apoptosis is the release of cytochrome c and a number of other proteins into the cytosol. In the cytoplasm, cytochrome c binds to the apoptotic protease-activating factor-1 (APAF-1) and ATP, forming an apoptosome. The apoptosome activates caspase-9, which in turn activates effector caspases-3 and -7, ultimately leading to apoptosis [1]. Furthermore, other soluble proteins released from mitochondria during apoptosis induction, such as Smac/DIABLO and the serine protease Omi/HtrA2, contribute to the caspase cascade by blocking inhibitors of apoptosis proteins (IAPs) and thus promoting caspase activation [1].

#### 3.1.2. Examples of LL-37-Induced Caspase-Dependent Apoptosis

The best-known example of the effect of LL-37 on the above-described mechanism of cell death is apoptosis of alveolar airway epithelial cells infected with P. aeruginosa. At physiologically relevant concentrations (10–30 μg/mL), LL-37 can induce Bax-dependent depolarization of the mitochondrial membrane in these cells, leading to cytochrome c release, subsequent activation of caspase-9 and caspase-3, and apoptosis. This type of caspase-dependent apoptosis occurs only in the presence of both the peptide and bacteria, but not with either stimulus alone [7]. Apoptosis of infected epithelial cells may represent an innate defense mechanism for the elimination of P. aeruginosa by the airway epithelium [40].

Lau et al. showed that LL-37 induces caspase-mediated cell death in lung epithelial cells (A549, 16HBE4o-) in vitro and in the airways of mice in vivo, with this effect being markedly reduced by a caspase-3 inhibitor [41].

It was also shown that LL-37 (30 μg/mL) increased caspase-3 levels and induced DNA fragmentation (at a concentration of 10 μg/mL) in cultured rat thoracic aortic smooth muscle cells, which has been associated with plasma membrane damage [8].

Studies on HSC-3 oral squamous cell carcinoma cells showed that stable expression of both C-terminally deleted LL-37 mutants and native LL-37 reduced the ability of these cells to form colonies, proliferate, migrate, and invade. The peptides induced caspase-3-mediated apoptosis via the p53-Bcl-2/Bax signaling pathway [42].

In addition to the caspase-dependent pathway, LL-37 is also capable of inducing caspase-independent cell death through other effector molecules.

### 3.2. Mitochondrial Caspase-Independent Apoptosis

Along with cytochrome c and Smac/DIABLO, apoptosis-inducing factor (AIF) and endonuclease G (EndoG) are also released through pores in the outer mitochondrial membrane, causing caspase-independent DNA fragmentation [1]. Caspase-independent apoptosis induced by LL-37 has been described predominantly in cancer cells.

#### 3.2.1. General Mechanism of Caspase-Independent Apoptosis

AIF is synthesized as a 62 kDa mitochondrial protein. Its activation requires cleavage by the cytosolic Ca^2+^-activated protease calpain I to generate a proapoptotic truncated form of 57 kDa [43]. Truncated AIF binds to cyclophilin A (CypA) in the cytosol and translocates to the nucleus [44], where it causes large-scale DNA fragmentation (20–50 kb) and chromatin condensation [1,6]. Endonuclease G (EndoG) is a mitochondrial, calpain-independent DNase that, following its release from the intermembrane space and translocation to the nucleus, efficiently degrades both single-stranded and double-stranded DNA into 10–190 bp fragments [45].

Calpains require intracellular Ca^2+^ for activation and can trigger apoptosis not only through AIF but also via proteolysis of cytoskeletal and membrane proteins [6]. LL-37 induces a rapid and sustained Ca^2+^ influx down its gradient from the extracellular space into the cytosol due to plasma membrane permeabilization and the formation of transmembrane pores [11]. Data indicate that Ca^2+^ and calpains are essential intermediate molecules in LL-37-induced apoptosis. Activated calpains are also involved in lysosomal permeabilization, leading to the release of proapoptotic lysosomal cathepsins and also causing Bax activation and mitochondrial destabilization following various stimuli [6]. Taken together, these findings suggest that LL-37 can activate multiple intermediary molecules and affect multiple organelles during the induction of apoptosis.

#### 3.2.2. Direct Action of LL-37 on the Mitochondrial Membrane

LL-37 is thought to accumulate on mitochondrial membranes and cause permeabilization of the outer mitochondrial membrane. In mitochondria isolated from MG63 cells, LL-37 releases AIF and cytochrome c, suggesting that LL-37 may directly interact with mitochondria, leading to dissipation of ΔΨm [46]. However, LL-37 alone is not sufficient to induce apoptosis, since activation and translocation of AIF, and hence activation of intracellular proteins following LL-37 exposure, are required [46]. This interaction affects only the outer mitochondrial membrane, as it leads to the release of AIF and cytochrome c, which are located in the intermembrane space, but not COX IV, which is localized on the inner membrane and is a well-known marker of mitochondrial integrity [46]. COX IV is retained in the mitochondria of human leukemia cells treated with a high concentration (40 μM) of LL-37 [6]. It is well-established that mitochondria originated from bacteria (the endosymbiotic theory), and consequently, both membranes contain a high proportion of anionic lipids. Therefore, LL-37 acts on the mitochondrial membrane in a manner similar to its action on the bacterial membrane, directly driven by this conserved lipid composition [6,46].

One study demonstrated that LL-37 at nanomolar concentrations specifically induces permeabilization of a biomimetic mitochondrial membrane rich in phosphoethanolamine (PE). PE lipids significantly facilitate the adsorption and accumulation of LL-37 on the PE-rich bilayer and also promote deeper insertion of peptide oligomers, particularly tetramers, into the bilayer. This work suggests an alternative pathway for LL-37-induced mitochondrial membrane permeabilization and apoptosis [47].

#### 3.2.3. Examples of LL-37-Induced Caspase-Independent Apoptosis

The most extensively characterized action of LL-37 regarding caspase-independent apoptosis is to promote cell death in malignant cells, as documented in several key studies [6,48,49,50]. In general terms, LL-37 treatment leads to a marked elevation in the levels of AIF and EndoG, alongside an increase in the proapoptotic proteins Bax and Bak. Concurrently, the concentration of the antiapoptotic protein Bcl-2 is decreased, reflecting a pattern of calpain-dependent apoptosis mediated by Bax activation [6,48,51]. Furthermore, LL-37 has been shown to upregulate the expression of both the transcription factor p53 and the PUMA protein [48,49].

The transcription factor p53 is among the primary responders to DNA damage, triggering DNA repair mechanisms and cell cycle arrest. It functions by binding to promoter regions that control the expression of several proapoptotic Bcl-2 family proteins. PUMA, which is a direct transcriptional target of p53, serves as a modulator of apoptosis in some cancer cell lines [52].

For instance, the death of the immortalized human T-cell line (Jurkat) induced by LL-37 (100–200 μg/mL) proceeds via a caspase-independent route. This process involves the calpain-dependent translocation of Bax to the mitochondria, a subsequent loss of ΔΨm, and the release and translocation of AIF to the nucleus. Although caspases were activated following LL-37 treatment (due to cytochrome c release), caspase inhibition did not affect LL-37-mediated apoptosis. In contrast, Ca^2+^ or calpain chelation or inhibition, as well as Bax/Bak knockout and Bcl-2 overexpression, inhibited cell death [6].

LL-37 (20–60 μM) promotes the translocation of AIF and EndoG from mitochondria to the nucleus in an in vivo mouse model of colon cancer (CRAMP^−/−^ mice (cathelicidin-deficient) with xenografts of human colon cancer cells) and human colon cancer cell lines (HCT116, HT-29) in vitro. This caspase-independent pathway may rely on p53-dependent activation of the proapoptotic proteins Bax and Bak, as well as the GPCR-mediated downregulation of Bcl-2 [3,48]. Furthermore, cathelicidin-deficient mice bearing colon tumors exhibited decreased apoptosis, elevated Bcl-2 expression, and lower p53-Bax/Bak expression, indicating that LL-37 initiates a signaling cascade involving GPCR, p53, and the Bax/Bak/Bcl-2 axis, ultimately leading to apoptosis via AIF/EndoG in colon cancer cells [3,48]. The same p53/Bax/Bcl-2 pathway is responsible for apoptosis in K562 leukemia cells upon treatment with Fe3O4 nanoparticles conjugated with the LL-37 peptide via an NH2 bridge [53].

In human osteosarcoma MG63 cells, LL-37 not only reduces viability but also triggers caspase-independent apoptosis [11,46]. Specifically, LL-37 is rapidly internalized, leading to the release of AIF into the cytosol and subsequently reducing the number and viability of MG63 cells in the micromolar concentration range with an IC50 value of approximately 5 μM. In osteoblasts, LL-37 induces an influx of extracellular Ca^2+^ into cells but triggers Ca^2+^-independent apoptosis [11].

LL-37 may also activate proapoptotic signaling without producing morphologically evident apoptosis. In Huh7 and HepG2 hepatocellular carcinoma cells, human cathelicidin (10–20 μM) decreased cell viability in a dose-dependent manner. In Huh7 cells, it specifically suppressed the cyclin D1–CDK4–p21 axis, thereby slowing the G1–S phase transition. Although LL-37 did not induce visible apoptosis, it enhanced the expression of proapoptotic genes (Bax, caspase-3, and caspase-8), suppressed the PI3K/Akt and MAPK/ERK (in HepG2 cells) survival pathways, and reduced the expression of genes encoding proinflammatory cytokines [54].

As an example in non-tumor cells, LL-37 at concentrations ≥ 10 μM induces apoptosis in urothelial cells. This effect occurs via a caspase-independent pathway, since no activated caspase-3 was detected and the levels of caspase-3 and caspase-7 remained unchanged, regardless of the LL-37 concentration [55].

### 3.3. Other Mechanisms of LL-37-Mediated Apoptosis

In addition to the “classical” mitochondrial pathways, LL-37 can induce apoptosis through mechanisms that are not directly related to disruption of mitochondrial membrane integrity. These include direct interaction with nuclear DNA, regulation of gene expression, and activation of the granzyme pathway in cytotoxic T lymphocytes.

It is known that human cathelicidin can actively interact with and bind to extracellular DNA and transport it into cells. For example, according to Sandgren et al., LL-37 associates with plasmid DNA, shielding it from degradation and facilitating its nuclear delivery in mammalian cells. This process relies on caveolae-independent, lipid raft-mediated endocytosis and requires cell surface proteoglycans [32]. Zuo et al. [56] demonstrated that the LL37-mtDNA complex (10 μg/mL LL37 + 2 μg/mL mtDNA) reduces cell viability, increases apoptosis, and promotes IL-1β/TNF-α release in LPS-treated rat alveolar epithelial cells (RLE-6TN). Additionally, LL37-mtDNA elevates ROS production and enhances the autophagy marker LC3B. The heat shock protein Hsp90aa1 was identified as a key downstream target; siRNA knockdown of Hsp90aa1 partially reversed these proapoptotic and pro-inflammatory effects. Thus, LL-37, when complexed with mtDNA, exacerbates epithelial cell injury via Hsp90aa1-dependent regulation of apoptosis, inflammation, and autophagy [56].

Moreover, LL-37 can translocate to the cell nucleus [57] and bind to gene promoter regions. Muñoz et al. demonstrated that LL-37 gene silencing in A375 melanoma cells affects the expression of genes involved in histone regulation, metabolic processes, stress responses, ubiquitination, and mitochondrial function [36].

A recent study showed that in addition to regulating promoters and gene expression, LL-37 binds to telomeric G-quadruplexes (G4) [35]. G4s are guanine-rich four-stranded DNA/RNA structures that form in telomeres and oncogene promoters (e.g., c-MYC, c-KIT, Bcl-2, VEGF). Their formation can block DNA polymerase activity, causing mutations, deletions, and genomic rearrangements, thereby contributing to tumor development. The cathelicidin LL-37 is capable of stabilizing telomeric DNA G-quadruplexes (G4) by binding to them and interfering with telomerase activity. Inhibition of telomerase-mediated telomere elongation may promote apoptosis in cancer cells. LL-37 binds to G-quadruplexes 10 times more efficiently than to double-stranded DNA [35].

Another mechanism by which LL-37 triggers apoptosis is granzyme-mediated apoptosis in cytotoxic T lymphocytes (CTLs). LL-37 at a concentration of 20 μg/mL induces the release of granzymes A and B (grA, grB) from intracellular CTL granules into the cytosol. GrA initiates caspase-independent apoptosis by acting on PARP-1 and its substrates Ku70 and NDUFS3, leading to ROS production, nuclear translocation of the SET complex, and subsequent single-stranded DNA breaks, p53 activation, and loss of cell membrane integrity. GrB, in contrast, activates caspase-dependent apoptosis by cleaving caspase-3, caspase-7, and BID [1,10].

Since the CTL cytosol contains the serpin PI-9, a potent inhibitor of grB [58], LL-37-induced apoptosis in these cells occurs predominantly via grA (caspase-independent pathway). However, in grB^−^/^−^ and grAB^−^/^−^ cells, caspase-dependent cell death is observed, possibly due to other granzymes such as grK [10].

### 3.4. LL-37 Derivatives and Apoptosis

Full-length LL-37 has several limitations that restrict its therapeutic use, including its relatively large size (37 amino acids), tendency to aggregate, rapid proteolytic degradation, moderate selectivity for cancer cells, and potential toxicity to normal tissues at high concentrations. Therefore, active research is underway on synthetic peptides—fragments and analogues of LL-37—that retain or even enhance the activity of the parent peptide, while often possessing better selectivity and a more predictable mechanism of action. The Table 1 summarizes data on selected LL-37 derivatives, their target cells, and the types and mechanisms of apoptosis they induce.

The table shows that several LL-37 derivatives and analogues exhibit antitumor activity, with some showing improved selectivity toward malignant cells compared with the parent peptide. Interestingly, although LL-37 promotes the survival and metabolic activity of breast cancer cells and is associated with the metastasis of estrogen-dependent tumors [68,69], its derivatives and analogues trigger caspase-dependent apoptosis in this type of cancer [49,64].

## 4. Antiapoptotic Effects of LL-37

Proapoptotic effects of LL-37 often involve membrane permeabilization and intracellular or mitochondrial targets, whereas antiapoptotic effects are predominantly associated with membrane receptor-mediated prosurvival signaling. Induction of apoptosis requires higher concentrations (typically 10–30 μg/mL and above), whereas antiapoptotic effects are observed at lower concentrations (0.01–5 μg/mL).

### 4.1. Inhibition of Apoptosis via Plasma Membrane Receptors

#### 4.1.1. Neutrophils: FPR2 and P2X7

LL-37 at concentrations of 0.01–5 μg/mL suppresses spontaneous neutrophil apoptosis by interacting with formyl peptide receptor 2 (FPR2) and the purinergic receptor P2X7 on the neutrophil surface, which modulate downstream signaling pathways [40,70].

FPR2 belongs to the class of Gi protein-coupled receptors, is activated by a wide range of structurally diverse pro- and anti-inflammatory ligands, and mediates the chemotactic response to LL-37 in neutrophils, monocytes, and T cells [24,71].

The human purinergic receptor P2X7 belongs to the family of ionotropic ATP-dependent receptors and is highly expressed on immune cells. Specific antagonists of the P2X7 signaling pathway have been shown to inhibit various functional effects of LL-37 [24]. LL-37 may influence P2X7 activity through binding involving the receptor’s transmembrane segments; alternatively, the peptide could induce non-lytic modifications of the surrounding plasma membrane without directly binding to the receptor [21].

Exposure of neutrophils to LL-37 increases the expression of the antiapoptotic protein Mcl-1, which inhibits mitochondrial damage and cytochrome c release and also inhibits the cleavage of BID by caspase-8 into tBID [40]. These effects block caspase-dependent mitochondrial apoptosis.

#### 4.1.2. Chronic Lymphocytic Leukemia (CLL) PBMC: CXCR4

LL-37 (5–10 μM) delays spontaneous apoptosis of leukemic cells by colocalizing with the CXCR4 receptor on the cell membrane. CXCR4 is a chemokine receptor crucial for the survival, migration, and homing of leukemic cells within the bone marrow microenvironment. High CXCR4 expression is associated with poor prognosis and an increased risk of relapse in acute myeloid leukemia (AML) and acute lymphoblastic leukemia (ALL) [72]. Interaction with LL-37 reduces CXCR4 expression on the cell surface, which may alter survival signals characteristic of CLL. This effect is confirmed by analysis of caspase-3 cleavage and increased expression of antiapoptotic Bcl-2 [73].

### 4.2. Survival Signaling Pathways Activated by LL-37

#### 4.2.1. PI3K/Akt and ERK1/2-MAPK Pathways

Inhibition of apoptosis by LL-37 in neutrophils is also mediated by PI3K activation and engagement of the PI3K/Akt/mTOR pathway. This pathway promotes the phosphorylation and inactivation of the proapoptotic proteins Bad and FoxO, enhances Bcl-2 synthesis, and activates the transcription factor NF-κB [40,70].

It was initially suggested that LL-37 exerts its antiapoptotic effect through the ERK1/2-MAPK pathway, inducing ERK1/2 phosphorylation and MAPK activation, as well as enhancing GM-CSF-dependent activation of this pathway, as occurs in primary monocytes. GM-CSF is also known to be a potent inhibitor of neutrophil apoptosis. ERK activation leads to increased expression of the antiapoptotic protein Bcl-XL and suppression of the proapoptotic protein tBID, which normally initiates mitochondrial damage and caspase-3 activation [74,75,76].

However, in neutrophils, activation of PI3K, rather than ERK1/2, plays a key role, in contrast to the effects of LL-37 in monocytes and epithelial cells [40]. Importantly, neutrophils in which LL-37 suppresses apoptosis retain the ability to respond to inflammatory stimuli, indicating the preservation of their functional competence [40].

These two pathways can be activated simultaneously. For example, in cardiomyocytes, LL-37 inhibits apoptosis by activating both the Akt and ERK1/2 pathways [13].

#### 4.2.2. COX-2/PGE2 Pathway

The COX-2/PGE2 pathway is a signaling cascade in which cyclooxygenase-2 (COX-2) converts arachidonic acid to prostaglandin H2, which is then converted to prostaglandin E2 (PGE2). PGE2 binds to EP2/EP4 receptors on the cell surface, activating prosurvival pathways such as PI3K/Akt and ERK/MAPK.

Via the COX-2/PGE2 pathway, LL-37 protects primary human keratinocytes and HaCaT cells from apoptosis induced by the topoisomerase I inhibitor camptothecin (CAM). In one study, LL-37 significantly reduced caspase-3 activity and increased COX-2 levels. Furthermore, LL-37 induced the expression of inhibitor of apoptosis protein 2 (IAP-2). A selective COX-2 inhibitor abolished the antiapoptotic effect of LL-37 and reduced IAP-2 expression, indicating that the antiapoptotic effect of LL-37 in keratinocytes is mediated by a COX-2-dependent mechanism involving IAP-2 [12].

Similarly, LL-37 inhibited sodium nitroprusside-induced apoptosis of dermal fibroblasts in systemic sclerosis (SSc): the peptide increased Bcl-2 and COX-2 expression, reduced Bax levels and caspase-3 activation, and stimulated PGE2 production, primarily through activation of the P2X7 receptor [14].

### 4.3. Indirect Inhibition of Apoptosis

LL-37 may inhibit apoptosis indirectly through its antimicrobial properties.

#### 4.3.1. Suppression of Virus-Induced Apoptosis

Upon infection with Coxsackievirus B3 (CVB3), which causes viral myocarditis and dilated cardiomyopathy, LL-37 inhibits CVB3 replication in primary cardiomyocytes and CVB3 transmission via exosomes without receptor interaction. LL-37 directly interacts with the exosomal heat shock protein 60 (HSP60) and blocks the diffusion of exosomes into recipient cells by destroying the exosomes and inhibiting HSP60-induced cardiomyocyte apoptosis [77].

#### 4.3.2. Inhibition of LPS-Induced Apoptosis

As part of its antibacterial function, LL-37 inhibits the binding of bacterial lipopolysaccharide to LPS receptors (CD14 and Toll-like receptor 4) on cells. Suppression of apoptosis by blocking LPS binding has been demonstrated in vitro and in vivo in human lung-derived microvascular endothelial cells (HMVEC-LBl), as well as in a mouse model of endotoxic shock, where LL-37 significantly suppressed apoptosis in liver endothelial cells and hepatocytes [78].

#### 4.3.3. Potential Association Between Endothelial-to-Mesenchymal Transition and Apoptosis Resistance

It was recently demonstrated that LL-37 induces endothelial-to-mesenchymal transition (EndMT), a process in which endothelial cells acquire a mesenchymal phenotype. EndMT is often associated with tissue fibrosis, inflammation, and resistance to apoptosis. Although the direct relationship between EndMT and the antiapoptotic effects of LL-37 remains to be fully elucidated, this finding expands our understanding of LL-37 as a multifunctional peptide capable of fundamentally altering the differentiation state and survival properties of cells [79].

Thus, the antiapoptotic effect of LL-37 is mediated through several complementary mechanisms: activation of membrane receptors (FPR2, P2X7, CXCR4), triggering of survival signaling pathways (PI3K/Akt, ERK1/2, COX-2/PGE2), and indirect cell protection via the antimicrobial properties of the peptide.

## 5. LL-37 and Other Types of Cell Death

In addition to apoptosis, LL-37 can regulate other forms of programmed cell death, such as pyroptosis, autophagy, and necrosis. These processes are closely linked to apoptotic pathways: they can share common signaling molecules (e.g., caspase-8), switch between one another depending on conditions, or occur simultaneously. Table 2 below summarizes examples of LL-37 involvement in the regulation of other types of cell death in various cellular models.

LL-37 inhibits LPS/ATP-induced pyroptosis in human macrophages, whereas under different experimental conditions it can promote pyroptotic/necrotic features in nasal epithelial cells and J774.2 macrophages. The cross-regulation between apoptosis, necrosis, pyroptosis, and autophagy mediated by LL-37 points to a complex network of signaling interactions that requires further investigation.

## 6. Discussion

It is evident that there is no single mechanism or universal pathway for LL-37-regulated apoptosis. The same applies to the antiapoptotic effect of cathelicidin. Among the factors that determine the different apoptotic pathways, the peptide concentration and the cell type can be identified.

Importantly, while plasma levels of LL-37 are typically around 1–2 μg/mL, local concentrations at sites of inflammation can be substantially higher. In psoriatic skin lesions, LL-37 concentrations may reach up to 300 μM (approximately 1.4 mg/mL). Such elevated local levels, generated by neutrophil degranulation and epithelial cell secretion, are well within the range shown to induce apoptosis in various cell types (10–30 μg/mL), supporting the pathophysiological relevance of the proapoptotic effects discussed below [89].

**Figure 2:**
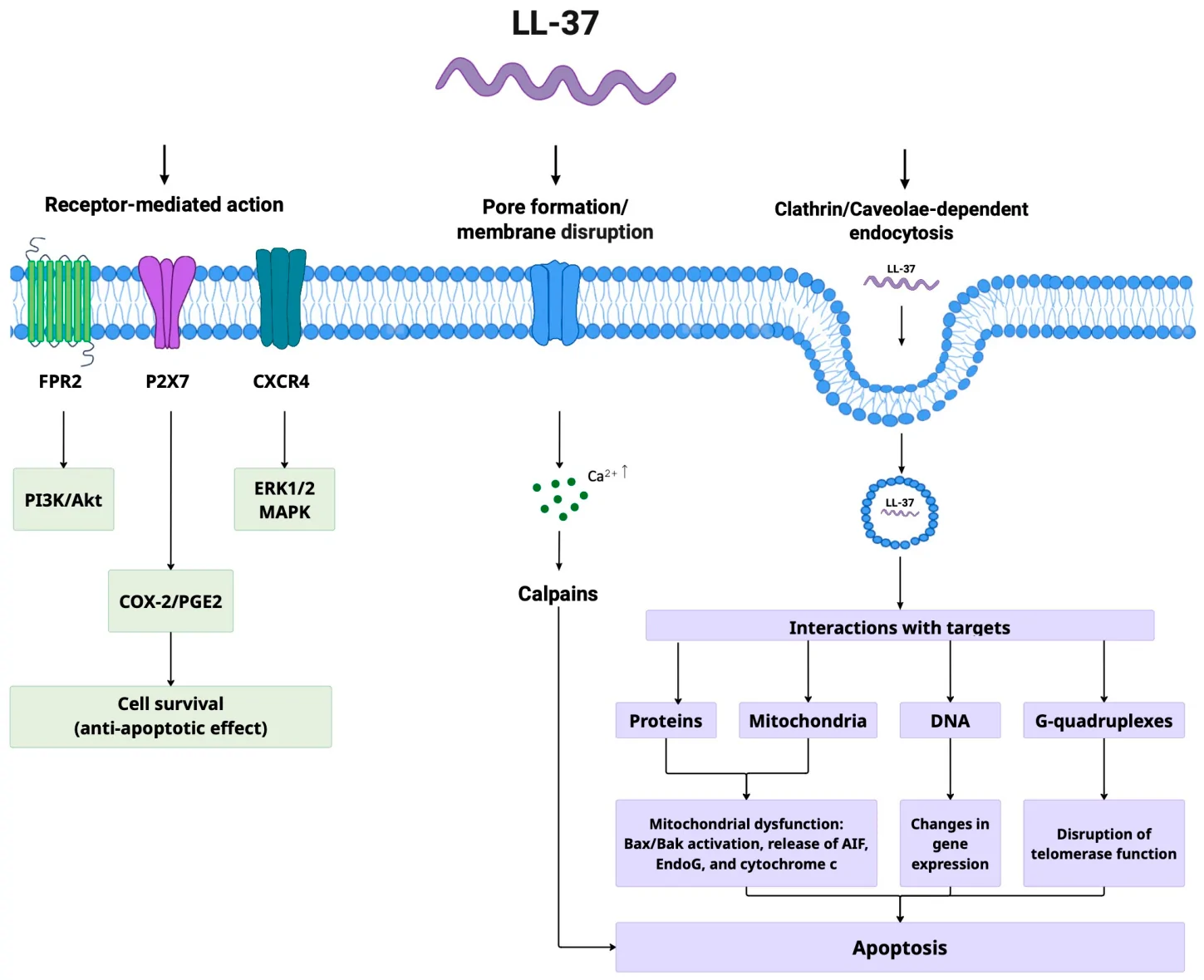
Context-dependent cellular mechanisms underlying the dual effects of LL-37 on apoptosis. The cellular effects of LL-37 depend on peptide concentration, cell type, membrane composition, receptor repertoire, and the cellular microenvironment. At relatively low concentrations, LL-37 primarily interacts with plasma membrane receptors, including FPR2, P2X7, and CXCR4, and activates cell type-specific prosurvival signaling pathways, such as PI3K/Akt, ERK1/2-MAPK, and COX-2/PGE2, thereby suppressing apoptosis. At higher concentrations, LL-37 can permeabilize the plasma membrane, increase cytosolic Ca^2+^ levels, activate calpains, and promote mitochondrial apoptosis. LL-37 can also enter cells through clathrin- and caveolae-dependent endocytosis and interact with intracellular proteins, mitochondrial membranes, gene regulatory regions, and telomeric G4. These interactions may alter gene expression, inhibit telomerase activity, activate Bax/Bak, and promote the release of cytochrome c, AIF, and EndoG. The depicted pathways are not mutually exclusive and may operate simultaneously or predominate under different cellular conditions.

Analysis of the literature suggests a general concentration-dependent trend in LL-37 activity, although substantial cell type- and context-dependent exceptions exist. The antiapoptotic effect occurs mainly at low concentrations (0.01–5 μg/mL) and is mediated by activation of membrane receptors (FPR2, P2X7, CXCR4) and survival signaling pathways (PI3K/Akt, ERK1/2, COX-2/PGE2). In contrast, the proapoptotic effect requires higher concentrations (10–30 μg/mL and above) and is typically mediated through the mitochondrial pathway—both caspase-dependent and caspase-independent. The proposed model integrating membrane receptor activation, plasma membrane permeabilization, intracellular uptake, and the resulting pro- or antiapoptotic effects of LL-37 is summarized in Figure 2.

For example, at 30 μg/mL, LL-37 induced apoptosis in vascular smooth muscle cells by disrupting cell membrane integrity and causing DNA fragmentation [8]. However, when comparing the induction of apoptosis and the blockade of proliferation, the concentration required to induce apoptosis was lower than that required to inhibit proliferation. Proapoptotic activity, as assessed by elevated caspase-3 levels, required 1 μM (4.55 μg/mL) LL-37, whereas the antiproliferative effect was observed at an LL-37 concentration of 8 μM (36.4 μg/mL) [9].

Neutrophils represent an interesting example of a complex response: LL-37 inhibits their apoptosis at low concentrations, induces it at higher concentrations, and at even higher concentrations (∼10 μg/mL after 4 h) triggers a switch from apoptosis to necrosis [40].

Interestingly, a recent study by Zhang et al. (2026) [90] demonstrated that LL-37 can form heteroaggregates with another AMP, human neutrophil peptide HNP-1. This heteroaggregation reduces the cytotoxicity of both peptides, and notably, LL-37 suppresses HNP-1-induced apoptosis in eukaryotic cells, whereas HNP-1 inhibits LL-37-induced necrosis in MDCK-I cells. These findings suggest that in the neutrophil microenvironment, interactions between different AMPs may fine-tune the balance between pro- and antiapoptotic signals, adding another layer of complexity to the regulation of cell death during inflammation.

LL-37 is capable of inducing both types of mitochondrial apoptosis. In general, lower proapoptotic concentrations (close to 10 μg/mL) are more often associated with the caspase-dependent pathway, whereas higher concentrations (≥20–40 μM) are associated with the caspase-independent pathway [91]. However, this rule is not absolute. In Jurkat T cells, LL-37 activates both pathways simultaneously, but caspase inhibition does not prevent cell death, indicating that the caspase-independent mechanism predominates [6].

Thus, a preliminary conclusion can be drawn. First, apoptosis requires supraphysiological concentrations of LL-37. Second, lower initiating concentrations are associated with caspase-dependent apoptosis, while higher concentrations are associated with the caspase-independent pathway.

A second major determinant of LL-37-mediated cell death is the simultaneous activation of multiple apoptotic mechanisms for certain cells or microorganisms, or the predominance of a particular apoptotic pathway depending on the conditions. For example, LL-37 induces apoptosis in C. albicans through multiple pathways. At nanomolar concentrations, it creates pores in the cell membrane, leading to ion efflux. LL-37 is then internalized into the cell and interacts with intracellular targets, promoting dissipation of the mitochondrial membrane potential, release of cytochrome c, and activation of the caspase cascade. Additionally, LL-37 releases Ca^2+^ from the endoplasmic reticulum, and mitochondrial Ca^2+^ uptake further exacerbates organelle dysfunction [92].

In regulatory T cells, LL-37 induces both caspase-dependent and caspase-independent apoptosis, suggesting that both processes can occur in this cell type [10]. In airway epithelial cells, LL-37 induces caspase-3 activation only upon bacterial infection and does not affect healthy cells [7]. Furthermore, recent studies have shown that the same GPCR-p53-Bax/Bak/Bcl-2 signaling pathway can be utilized by LL-37 to induce apoptosis in both a caspase-independent and a caspase-dependent manner [42,65]. Apparently, further research is needed to understand the mechanisms underlying the choice of the apoptotic pathway upon LL-37 exposure.

The dependence of the apoptotic effects of LL-37 on cell type becomes even more evident when considering the interaction of LL-37 with tumor cells. Several examples illustrating the interaction of LL-37 with various cancer types are given in Table 3.

Human cathelicidin exhibits a paradoxical, tumor-type- and context-dependent dual role. In some malignancies, LL-37 promotes proliferation, migration, invasion, or cell survival, whereas in others it suppresses tumor growth or promotes apoptotic signaling. These differences appear to reflect the receptor repertoire, intracellular signaling state, p53 status, membrane properties, peptide concentration, and tumor microenvironment. Representative examples are summarized in Table 3.

In antitumor and proapoptotic settings, LL-37 can act through several mechanisms, including proteasome inhibition, p53-dependent signaling, suppression of survival pathways, direct mitochondrial interactions, and release of AIF and EndoG. In gastric and colon cancer, as well as osteosarcoma, these effects frequently involve intracellular or mitochondrial mechanisms rather than classical receptor-mediated prosurvival signaling.

In contrast, pro-tumorigenic effects of LL-37 are frequently associated with receptor- or membrane-mediated signaling involving pathways such as PI3K/Akt, MAPK/ERK, STAT3, NF-κB, and β-catenin. Depending on the tumor type, these pathways may promote proliferation, survival, EMT, migration, invasion, or the establishment of an immunosuppressive tumor microenvironment.

In certain cancer types (melanoma, pancreatic cancer), LL-37 can both stimulate and inhibit cell death in a dose-dependent manner. At low concentrations, the receptor-mediated survival response predominates; at high concentrations, direct mitochondrial damage, increased ROS, and a switch of the immune microenvironment toward anti-tumor immunity prevail. Thus, the outcome in such cases is determined by the local concentration of the peptide. Notably, LL-37 derivatives (FK-16, KR12C, 17BIPHE2, KI-21-3), which bypass receptor-mediated survival cascades, often exhibit a more pronounced pro-apoptotic effect than the native peptide and act even in those cancer types where LL-37 exerts an anti-apoptotic effect (e.g., in breast cancer). This opens up prospects for the design of selective peptide analogues.

The majority of studies focus on the effects of exogenous LL-37; however, interesting results have been obtained by reducing the expression of the endogenous peptide. For example, methylation of the CAMP gene promoter in chondrocytes in osteoarthritis increases TGF-β expression, reduces chondrocyte proliferation and inflammatory responses, thereby stimulating their apoptosis and indirectly confirming the antiapoptotic function of endogenous LL-37 in cartilage tissue [110]. In colorectal cancer, LL-37 expression is significantly reduced in the tumor compared to normal tissue, and cathelicidin-knockout mice develop more and larger tumors [48,111]. In these tumors, the expression of p53, Bax, and Bak is reduced, while Bcl-2 expression is increased, accompanied by a decreased basal level of apoptosis [48]. Similar data have been obtained for hepatocellular carcinoma [54] and oral squamous cell carcinoma [42]. Thus, suppression of endogenous LL-37 is associated with cancer progression and reduced apoptosis, indicating a proapoptotic and anti-tumor role of the peptide in certain types of cancer.

In addition to apoptosis, LL-37 regulates other cell-death pathways and cell-survival processes, including pyroptosis, necrosis, and autophagy. These processes share common signaling molecules with apoptotic pathways. For example, caspase-8 plays a central role in extrinsic apoptosis and serves as a molecular switch between apoptosis, necroptosis, and pyroptosis. LL-37 induces inflammatory cell death (pyroptosis/necrosis) accompanied by a significant increase in caspase-8 activity, while caspases-3 and -7 remain inactive [83]. At the same time, caspase-8 can participate in the intrinsic pathway of apoptosis by cleaving BID to tBID [112]. Another example is the suppression of autophagy induced by LL-37 via mTOR activation, which leads to a decrease in mitochondrial membrane potential and accumulation of ROS, as well as subsequent DNA damage in pancreatic cancer cells [99]. Understanding how LL-37 switches between different types of cell death remains an important task for future research.

Finally, despite the abundance of accumulated data, a number of questions remain open. For example, why does LL-37 suppress apoptosis in some cell types, such as neutrophils, while promoting apoptosis in others, including infected airway epithelial cells? Or, how does the choice between triggering caspase-dependent versus caspase-independent apoptosis occur upon activation of the same signaling pathway? Further investigation of the molecular mechanisms underlying these opposing effects is necessary to realize the therapeutic potential of LL-37 and its derivatives in oncology, immunology, and regenerative medicine.

## 7. Future Perspectives

There are several promising directions for further research and potential applications of LL-37 in the regulation of cell death. First, LL-37 may serve as a promising tool for the selective elimination of cytotoxic T lymphocytes (CTLs) in autoimmune diseases. In contrast to existing non-specific immunosuppressive approaches associated with numerous side effects, the proapoptotic effect of LL-37 on CTLs would allow targeted modulation of the immune response. Furthermore, uncovering the mechanism by which neutrophils attenuate CTL activity via LL-37 opens new avenues for understanding the cross-regulation between innate and adaptive immunity.

A key remaining challenge is to decipher the mechanisms that determine whether LL-37 exerts pro- or antiapoptotic effects in a given cellular context. In particular, it remains unclear why LL-37 suppresses apoptosis in neutrophils yet promotes the death of infected airway epithelial cells. We hypothesize that these divergent outcomes are determined by differences in receptor repertoire and stoichiometry, membrane composition, adaptor recruitment, and the spatiotemporal dynamics of downstream signaling. CRISPR/Cas9-mediated perturbation of candidate signaling regulators, including β-arrestin-1/2, Gab2, PI3Kγ, and p53, could help define their contribution to the apoptotic outcome, while bioluminescence resonance energy transfer (BRET) can be used to visualize receptor–adaptor complexes and quantify their formation dynamics in living cells.

Another critical question concerns the switch between caspase-dependent and caspase-independent apoptosis upon activation of seemingly similar signaling pathways. LL-37 induces caspase-dependent apoptosis in infected airway epithelial cells and HSC-3 oral carcinoma cells, yet triggers caspase-independent death via AIF and EndoG in Jurkat, colorectal cancer and osteosarcoma cells. This dichotomy may be governed by the kinetics and amplitude of outer mitochondrial membrane permeabilization: rapid, extensive permeabilization releases both cytochrome c and AIF, but when cytochrome c release is insufficient to assemble functional apoptosomes—for instance, due to overexpression of apoptosis-inhibitory proteins—the AIF-mediated caspase-independent route prevails. To test this hypothesis, we propose live-cell imaging with fluorescent reporters for cytochrome c and AIF to quantify their release kinetics from mitochondria in real time. Pharmacological and genetic modulation of mitochondrial membrane permeabilization, using Bax/Bak-targeting compounds and Bcl-2 overexpression, respectively, could help define the balance between the two pathways. Finally, investigating the role of calpains activated by LL-37-induced Ca^2+^ influx may clarify why AIF activation and caspase degradation predominate in certain cell types.

An additional important direction is the use of the proapoptotic activity of LL-37 to combat viral infections. Given that some viruses (e.g., human herpesvirus 8 and molluscum contagiosum virus) express apoptosis-inhibitory proteins such as v-FLIP (which mimics cellular FLIP and blocks death receptor signaling) [113,114] or cytomegalovirus vMIA (which binds and sequesters the proapoptotic protein Bax at mitochondria) [115], LL-37 might provide a means of engaging alternative cell-death mechanisms that are less sensitive to specific viral antiapoptotic strategies. By inducing caspase-dependent apoptosis or alternative cell death pathways, LL-37 can eliminate infected cells that the virus attempts to protect from apoptosis, opening up prospects for the development of antiviral therapies based on it.

In oncology, enhancing the proapoptotic potential of LL-37 itself through delivery technologies may be promising: immobilization of LL-37 on the surface of magnetic nanoparticles significantly increases its anti-tumor activity, as demonstrated in DLD-1 and HT-29 colon cancer cell lines [116]. Tissue-specific delivery would avoid the problems associated with the dual role of cathelicidin in tumor pathogenesis.

Another approach could be the use of anti-LL-37 antibodies for those cancer types in which LL-37 exhibits antiapoptotic activity, thereby reducing cancer cell survival and enhancing the action of CTLs in the tumor microenvironment. Thus, by modulating the apoptotic function of LL-37, novel therapeutic approaches for a wide range of diseases can be developed.

## 8. Conclusions

LL-37 exhibits a dual ability to both induce and suppress apoptosis. Its proapoptotic action is mediated primarily through the mitochondrial pathway and can occur in either a caspase-dependent (with the release of cytochrome c and the activation of caspases-9 and -3) or a caspase-independent manner, via the translocation of the apoptosis-inducing factor AIF and endonuclease G to the nucleus. Key mediators of this proapoptotic action include calpains, Ca^2+^ signaling, Bcl-2 family proteins, particularly Bax and Bak, and the transcription factor p53. At the same time, LL-37 can suppress apoptosis in various cell types by activating plasma membrane receptors and prosurvival pathways such as PI3K/Akt, ERK1/2, and COX-2/PGE2, as well as by increasing the expression of antiapoptotic proteins including Mcl-1 and Bcl-2. The selection of a particular apoptotic pathway and the balance between pro- and antiapoptotic effects are determined by multiple factors, including LL-37 concentration, cell type, receptor repertoire, and the cellular microenvironment.

## Figures and Tables

**Table 1:** Apoptotic effect of some synthetic derivatives and analogues of LL-37.

Derivative	Total Length (aa)	Net Electrical Charge at pH 7	Cells	Concentration	Mechanism
hCAP18 109–135 (C-terminal domain of LL-37) * FRKSKEKIGKEFKRIVQRIKDFLRNLV Analogues: FF/CAP18 FRKSKEKIGKFFKRIVQRIFDFLRNLV LL/CAP18 FRKSKEKIGKLFKRIVQRILDFLRNLV	27 27 27	+7 +6 +7	SAS-H1 (oral cavity cancer) Healthy human gingival fibroblasts (HGF) Human keratinocyte cell line HaCaT	5–40 μg/mL, dose-dependent effect	Caspase-independent mitochondrial apoptosis in SAS-H1 cells; no significant cytotoxicity toward HGF or HaCaT cells. Induction of apoptosis in SAS-H1 and HGF cells, with moderate cytotoxicity toward HaCaT cells [59]
FF/CAP18 FRKSKEKIGKFFKRIVQRIFDFLRNLV	27	+6	HCT116	10–40 μg/mL	Caspase-independent mitochondrial apoptosis, p53-independent [60,61]
FK-16 FKRIVQRIKDFLRNLV	16	+4	LoVo, HCT116 (colon cancer)	20–40 μM	GPCR → p53 → Bax/Bak/AIF/EndoG pathway Caspase-independent mitochondrial apoptosis + autophagy [62]
17BIPHE2 GBKRLVQRLKDBLRNLV, where B is biphenylalanine	17	+4	A549 (lung cancer) Healthy lung epithelial cells BEAS-2B and NCI-H1795	25–35 μM	Mitochondrial apoptosis via the ERK ** pathway and regulation of Bax and Bcl-2 expression. Non-toxic to BEAS-2B and NCI-H1795. [63]
KR12C KRIVKLIKKWLR	12	+7	MCF-7 (breast cancer)	5–10 μM	Suppression of the c-Myc promoter via binding to G-quadruplexes. Disruption of the VEGF-A-Bcl-2 pathway. Increased levels of p53, PARP, caspase-8 and APAF-1. Caspase-dependent apoptosis. [64]
KI-21-3 KIGKFFKRIVRIKKFIRKFV-NH_2_	21	+10	SCC-4 (oral cavity cancer)	5 μg/kg per mouse	Caspase-dependent apoptosis in vivo [65]
CSA-13 ceragenin, a non-peptide amphipathic analogue of LL-37 in its free form MNP@CSA-13 (a set of ceragenin molecules immobilized on magnetic particles)	- -	+4 High positive charge	MCF-7 MDA-MB-231 (breast cancer)	10–20 μg/mL	Caspase-dependent apoptosis [49]

* Amino acid sequence of native LL-37 is LLGDFFRKSKEKIGKEFKRIVQRIKDFLRNLVPRTES. ** ERK is a serine/threonine protein kinase widely distributed in eukaryotic cells and belongs to the MAPK protein family. The ERK signaling pathway influences cell proliferation, invasion, and migration [6]. A study showed that phosphorylated (p-)ERK can mediate apoptosis in pancreatic cancer cells by regulating the Bax/Bcl-2 ratio [66]. Studies have also shown that ERK controls apoptosis in colorectal tumor cells by regulating the expression of Bax and Bcl-2 [6,67].

**Table 2:** Examples of regulation of different types of cell death by LL-37.

Cell Death Type	Cell Type	LL-37 Effects	Mechanism of Action	References
Pyroptosis	Human macrophages	Inhibition	Inhibits the LPS binding/ATP/P2X7-induced IL-1β expression, caspase-1 activation and inflammasome formation	[80,81]
	Alveolar epithelium (cell line A549)	Inhibition	Downregulation of expression of NLRP3, caspase-1 p20, NT-GSDMD, IL-18 and IL-1β	[82]
Pyroptosis/necrosis	Human nasal epithelial cells (HNEpCs) and mouse macrophages (J774.2)	Induction	Activation of caspases-1 and -8, caspases-3 and -7 are inactive. Increased ATP secretion from both cell types and increased IL-6 and IL-8 release from HNEpCs. Rapid necrotic morphological changes.	[83]
Autophagy	Endothelial cells (HUVEC)	Induction	Upregulation of LC3-II expression (autophagosomal membrane marker) in the cells with the dysfunction of autophagy and delayed intracellular degradation of LL-37	[84]
	Human colon cancer cell lines (LoVo and HCT116)	Induction by FK-16	Enhanced expression of LC3-I/II, Atg5 and Atg7 and increased formation of LC3-positive autophagosomes.	[62]
	Human keratinocytes HaCaT	Induction	Activation of transcription factor EB (TFEB), increased nuclear translocation of TFEB, and increased expression of ATG5, ATG7, and beclin 1 (BECN1)	[85]
	Macrophages	Induction	Macrophage-derived LL-37, but not neutrophil-derived LL-37, induces autophagy. The native form, the N-terminal dileucine motif of LL-37 and intracellular DPP-1 activity in the target cells are required.	[86]
Necrosis	Neutrophils	Induction	Secondary necrosis with release of IL-8, IL-1R receptor antagonist, ATP and intact neutrophilic granules	[87,88]

**Table 3:** Molecular Mechanisms of Pro- and Anti-Apoptotic Effects of LL-37 in Different Cancer Types.

Effect	Cancer Type	Main Receptors/Interactions	Key Signaling Pathways and Molecular Mechanisms	References
Proapoptotic	Gastric cancer	• Proteasome inhibition • BMP receptor II • p53 activation	• Inhibition of chymotrypsin-like and caspase-like activity of the 20S proteasome → BMP4 accumulation → BMP receptor II activation → Smad1/5 phosphorylation → p21 activation → G1–S cell-cycle arrest and growth suppression • ↑Bax, ↑PUMA, ↑cleaved caspase-3 → ↓Bcl-2 → apoptosis	[93,94]
Proapoptotic	Colon cancer (LL-37 and FF/CAP18 derivative)	• GPCR (pertussis toxin-sensitive) • Direct interaction with the mitochondrial membrane • Effect on microRNAs (miRNAs)	• GPCR binding → p53 activation → ↑Bax/↑Bak → ↓Bcl-2 → caspase-independent apoptosis • Release of AIF and EndoG from mitochondria → nuclear translocation → caspase-independent apoptosis • ↑miR-663a → ↓CXCR4 → ↓Akt phosphorylation → p21 activation → G2/M cell-cycle arrest	[48,95]
Proapoptotic	Osteosarcoma	• PTEN/Akt/mTOR pathway • Direct interaction with the mitochondrial membrane	• Suppression of SQLE-mediated cholesterol synthesis through the PTEN/Akt/mTOR–SREBP2/SQLE axis • Direct mitochondrial effects with AIF release and caspase-independent apoptosis • Induction of apoptosis and pyroptotic signaling	[46,51,96]
Dual	Melanoma (keratinocyte-derived LL-37)	• P2X7 (pathway blockade)	• Pro-tumorigenic at low doses (≤40 μg/mL): ↑YB-1 expression → NF-κB pathway activation → tumor progression • Anti-tumorigenic at high doses (40–80 μg/mL): P2X7 blockade → ↓PI3K/Akt → EMT suppression • Formation of an antitumor immune microenvironment (M1 macrophage polarization, N1 neutrophil polarization) → inhibition of viability and migration	[97,98]
Dual	Pancreatic cancer	• FPR2 (FPRL1) • P2X7 • CXCR4 • TLR2	• Pro-tumorigenic at low doses (≤1 μM; tumor microenvironment): FPR2/P2X7 activation → ↑pluripotency-associated gene expression → ↑self-renewal and invasion of cancer stem cells; CXCR4 stabilization • Anti-tumorigenic at high doses (>2 μM): mTOR pathway activation → suppression of autophagy → mitochondrial dysfunction → ↑ROS → DNA breakage → cell-cycle arrest • Reprogramming of the tumor immune microenvironment (↓MDSCs, ↓M2 macrophages, ↑CD8^+^/CD4^+^ T cells)	[99,100]
Anti-apoptotic	Hepatocellular carcinoma	• HER2/EGFR transactivation • TAM-mediated immunosuppression	• In Huh7 and HepG2 cells, LL-37 reduces viability, enhances proapoptotic gene expression, and suppresses PI3K/Akt and MAPK/ERK signaling. • In other hepatocellular carcinoma models, LL-37 promotes HER2/EGFR-associated MAPK/ERK signaling, EMT, migration, and metastasis.	[54,101]
Anti-apoptotic	Prostate cancer	• FPR2 (FPRL1; autocrine/paracrine)	• LL-37 overexpression in primary and metastatic prostate cancer • FPR2 activation → ↑M-CSF and MCP-1 via NF-κB and STAT3 → recruitment of immature macrophages and differentiation toward M2 TAMs • Autocrine signaling regulates pro-tumorigenic chemokines (MCP-1, CXCL1/2)	[102,103]
Anti-apoptotic	Bladder cancer	• Sphingosine-1-phosphate (S1P) receptor-mediated signaling	• S1P stimulates hCAP18/LL-37 expression and release from bladder cancer cells • LL-37 dose-dependently increases proliferation of bladder cancer cells • Paracrine mechanism: S1P from the inflammatory microenvironment → LL-37 production → tumor growth promotion	[104]
Anti-apoptotic	Breast cancer	• TRPV2 and BKCa (calcium channels) • Membrane glycan binding (syndecan-4, GAGs) • CXCR4	• Syndecan-4/GAG-dependent membrane binding → PI3K/Akt activation → ↑intracellular Ca^2+^ → TRPV2/BKCa activation → stimulation of migration and invasion • CXCR4-dependent enhancement of metastatic behavior • EMT induction (↑N-cadherin, ↓γ-catenin)	[15,68,105,106]
Anti-apoptotic	Lung cancer	• TLR4 • EGFR	• TLR4 binding → Akt and GSK3β phosphorylation → β-catenin accumulation → nuclear translocation → transcription of Wnt/β-catenin target genes (cyclin D1, c-Myc) • Pro-tumorigenic at low doses (5 ng/mL): EGFR phosphorylation → activation of MAPK signaling pathways → increased proliferation and growth	[107,108]
Anti-apoptotic	Ovarian cancer	• FPR2 (FPRL1)	• FPR2 binding → sustained MAPK/ERK activation → CREB phosphorylation • ↑MMP-2 and MMP-9 secretion → enhanced invasion	[109]
Anti-apoptotic	Chronic lymphocytic leukemia	• CXCR4	• LL-37 binding to CXCR4 → ↓surface CXCR4 expression → delayed spontaneous apoptosis • ↑Bcl-2 expression	[73]

Symbols: ↑, increased expression, activity, or level; ↓, decreased expression, activity, or level; →, downstream mechanistic consequence or progression of the indicated pathway.

## Data Availability

No new data were created or analyzed in this study. Data sharing is not applicable to this article.

## Abbreviations

The following abbreviations are used in this manuscript:

AIF	apoptosis-inducing factor
Akt	protein kinase B
Bak	Bcl-2 homologous antagonist/killer
Bax	Bcl-2-associated X protein
Bcl-2	B-cell lymphoma 2
BID	BH3-interacting domain death agonist
COX-2	cyclooxygenase-2
CTL	cytotoxic T lymphocyte
CVB3	Coxsackievirus B3
CXCR4	C-X-C motif chemokine receptor 4
EndMT	endothelial-to-mesenchymal transition
EndoG	endonuclease G
ERK	extracellular signal-regulated kinase
ERK1/2	extracellular signal-regulated kinases 1 and 2
FPR2	formyl peptide receptor 2
G4	G-quadruplex
GPCR	G protein-coupled receptor
grB	granzyme B
HGF	human gingival fibroblasts
HNP-1	human neutrophil peptide 1
Hsp90aa1	heat shock protein 90 alpha family class A member 1
IAP-2	inhibitor of apoptosis protein 2
IL-1β	interleukin 1 beta
LC3	microtubule-associated protein 1 light chain 3
LL-37	human cathelicidin LL-37
LPS	lipopolysaccharide
MAPK	mitogen-activated protein kinase
mtDNA	mitochondrial DNA
P2X7	purinergic receptor P2X7
p53	tumor protein p53
PE	phosphoethanolamine
PGE2	prostaglandin E2
PI3K	phosphatidylinositol 3-kinase
ROS	reactive oxygen species
tBID	truncated BID
ΔΨm	mitochondrial membrane potential

## References

[1] Mustafa M., Ahmad R., Tantry I.Q., Ahmad W., Siddiqui S., Alam M., Abbas K., Moinuddin, Hassan M.I., Habib S. (2024). Apoptosis: A Comprehensive Overview of Signaling Pathways, Morphological Changes, and Physiological Significance and Therapeutic Implications. Cells.

[2] Lehrer R.I., Ganz T. (2002). Cathelicidins: A Family of Endogenous Antimicrobial Peptides. Curr. Opin. Hematol..

[3] Keshri A.K., Rawat S.S., Chaudhary A., Sharma S., Kapoor A., Mehra P., Kaur R., Mishra A., Prasad A. (2025). LL-37, the Master Antimicrobial Peptide, Its Multifaceted Role from Combating Infections to Cancer Immunity. Int. J. Antimicrob. Agents.

[4] Johansson J., Gudmundsson G.H., Rottenberg M.E., Berndt K.D., Agerberth B. (1998). Conformation-Dependent Antibacterial Activity of the Naturally Occurring Human Peptide LL-37. J. Biol. Chem..

[5] Oren Z., Lerman J.C., Gudmundsson G.H., Agerberth B., Shai Y. (1999). Structure and Organization of the Human Antimicrobial Peptide LL-37 in Phospholipid Membranes: Relevance to the Molecular Basis for Its Non-Cell-Selective Activity. Biochem. J..

[6] Mader J.S., Mookherjee N., Hancock R.E.W., Bleackley R.C. (2009). The Human Host Defense Peptide LL-37 Induces Apoptosis in a Calpain- and Apoptosis-Inducing Factor–Dependent Manner Involving Bax Activity. Mol. Cancer Res..

[7] Barlow P.G., Beaumont P.E., Cosseau C., Mackellar A., Wilkinson T.S., Hancock R.E.W., Haslett C., Govan J.R.W., Simpson A.J., Davidson D.J. (2010). The Human Cathelicidin LL-37 Preferentially Promotes Apoptosis of Infected Airway Epithelium. Am. J. Respir. Cell Mol. Biol..

[8] Ciornei C.D., Tapper H., Bjartell A., Sternby N.H., Bodelsson M. (2006). Human Antimicrobial Peptide LL-37 Is Present in Atherosclerotic Plaques and Induces Death of Vascular Smooth Muscle Cells: A Laboratory Study. BMC Cardiovasc. Disord..

[9] Jönsson D., Nilsson B.-O. (2012). The Antimicrobial Peptide LL-37 Is Anti-inflammatory and Proapoptotic in Human Periodontal Ligament Cells. J. Periodontal Res..

[10] Mader J.S., Ewen C., Hancock R.E.W., Bleackley R.C. (2011). The Human Cathelicidin, LL-37, Induces Granzyme-Mediated Apoptosis in Regulatory T Cells. J. Immunother..

[11] Säll J., Carlsson M., Gidlöf O., Holm A., Humlén J., Öhman J., Svensson D., Nilsson B.-O., Jönsson D. (2013). The Antimicrobial Peptide LL-37 Alters Human Osteoblast Ca^2+^ Handling and Induces Ca^2+^-Independent Apoptosis. J. Innate Immun..

[12] Chamorro C.I., Weber G., Grönberg A., Pivarcsi A., Ståhle M. (2009). The Human Antimicrobial Peptide LL-37 Suppresses Apoptosis in Keratinocytes. J. Investig. Dermatol..

[13] Miao S., Liu H., Yang Q., Zhang Y., Chen T., Chen S., Mao X., Zhang Q. (2024). Cathelicidin Peptide LL-37: A Multifunctional Peptide Involved in Heart Disease. Pharmacol. Res..

[14] Kim H.J., Cho D.H., Lee K.J., Cho C.S., Bang S.I., Cho B.K., Park H.J. (2011). LL-37 Suppresses Sodium Nitroprusside-Induced Apoptosis of Systemic Sclerosis Dermal Fibroblasts: Letter to the Editor. Exp. Dermatol..

[15] Habes C., Weber G., Goupille C. (2019). Sulfated Glycoaminoglycans and Proteoglycan Syndecan-4 Are Involved in Membrane Fixation of LL-37 and Its Pro-Migratory Effect in Breast Cancer Cells. Biomolecules.

[16] Sharma B., Kanwar S.S. (2018). Phosphatidylserine: A Cancer Cell Targeting Biomarker. Semin. Cancer Biol..

[17] Capozzi E., Aureli S., Minicozzi V., Rossi G.C., Stellato F., Morante S. (2018). Designing Effective Anticancer-Radiopeptides. A Molecular Dynamics Study of Their Interaction with Model Tumor and Healthy Cell Membranes. Biochim. Biophys. Acta Biomembr..

[18] Yang D., Chen Q., Schmidt A.P., Anderson G.M., Wang J.M., Wooters J., Oppenheim J.J., Chertov O. (2000). Ll-37, the Neutrophil Granule–And Epithelial Cell–Derived Cathelicidin, Utilizes Formyl Peptide Receptor–Like 1 (Fprl1) as a Receptor to Chemoattract Human Peripheral Blood Neutrophils, Monocytes, and T Cells. J. Exp. Med..

[19] Subramanian H., Gupta K., Guo Q., Price R., Ali H. (2011). Mas-Related Gene X2 (MrgX2) Is a Novel G Protein-Coupled Receptor for the Antimicrobial Peptide LL-37 in Human Mast Cells. J. Biol. Chem..

[20] Zhang Z., Cherryholmes G., Chang F., Rose D.M., Schraufstatter I., Shively J.E. (2009). Evidence That Cathelicidin Peptide LL-37 May Act as a Functional Ligand for CXCR2 on Human Neutrophils. Eur. J. Immunol..

[21] Tomasinsig L., Pizzirani C., Skerlavaj B., Pellegatti P., Gulinelli S., Tossi A., Virgilio F.D., Zanetti M. (2008). The Human Cathelicidin LL-37 Modulates the Activities of the P2X7 Receptor in a Structure-Dependent Manner. J. Biol. Chem..

[22] Scheenstra M.R., van Harten R.M., Veldhuizen E.J.A., Haagsman H.P., Coorens M. (2020). Cathelicidins Modulate TLR-Activation and Inflammation. Front. Immunol..

[23] Tjabringa G.S., Aarbiou J., Ninaber D.K., Drijfhout J.W., Sørensen O.E., Borregaard N., Rabe K.F., Hiemstra P.S. (2003). The Antimicrobial Peptide LL-37 Activates Innate Immunity at the Airway Epithelial Surface by Transactivation of the Epidermal Growth Factor Receptor. J. Immunol..

[24] Verjans E.-T., Zels S., Luyten W., Landuyt B., Schoofs L. (2016). Molecular Mechanisms of LL-37-Induced Receptor Activation: An Overview. Peptides.

[25] Burton M.F., Steel P.G. (2009). The Chemistry and Biology of LL-37. Nat. Prod. Rep..

[26] Shahmiri M., Enciso M., Adda C.G., Smith B.J., Perugini M.A., Mechler A. (2016). Membrane Core-Specific Antimicrobial Action of Cathelicidin LL-37 Peptide Switches Between Pore and Nanofibre Formation. Sci. Rep..

[27] Sancho-Vaello E., Gil-Carton D., François P., Bonetti E.-J., Kreir M., Pothula K.R., Kleinekathöfer U., Zeth K. (2020). The Structure of the Antimicrobial Human Cathelicidin LL-37 Shows Oligomerization and Channel Formation in the Presence of Membrane Mimics. Sci. Rep..

[28] Kilari G., Tran J., Blyth G.A.D., Cobo E.R. (2025). Human Cathelicidin LL-37 Rapidly Disrupted Colonic Epithelial Integrity. Biochim. Biophys. Acta Biomembr..

[29] Tang X., Basavarajappa D., Haeggström J.Z., Wan M. (2015). P2X7 Receptor Regulates Internalization of Antimicrobial Peptide LL-37 by Human Macrophages That Promotes Intracellular Pathogen Clearance. J. Immunol..

[30] Tokajuk J., Deptuła P., Piktel E., Daniluk T., Chmielewska S., Wollny T., Wolak P., Fiedoruk K., Bucki R. (2022). Cathelicidin LL-37 in Health and Diseases of the Oral Cavity. Biomedicines.

[31] Xhindoli D., Pacor S., Benincasa M., Scocchi M., Gennaro R., Tossi A. (2016). The Human Cathelicidin LL-37—A Pore-Forming Antibacterial Peptide and Host-Cell Modulator. Biochim. Biophys. Acta Biomembr..

[32] Sandgren S., Wittrup A., Cheng F., Jönsson M., Eklund E., Busch S., Belting M. (2004). The Human Antimicrobial Peptide LL-37 Transfers Extracellular DNA Plasmid to the Nuclear Compartment of Mammalian Cells via Lipid Rafts and Proteoglycan-Dependent Endocytosis. J. Biol. Chem..

[33] Suzuki K., Murakami T., Hu Z., Tamura H., Kuwahara-Arai K., Iba T., Nagaoka I. (2016). Human Host Defense Cathelicidin Peptide LL-37 Enhances the Lipopolysaccharide Uptake by Liver Sinusoidal Endothelial Cells without Cell Activation. J. Immunol..

[34] Zhang Z., Le K., La Placa D., Armstrong B., Miller M.M., Shively J.E. (2020). CXCR2 Specific Endocytosis of Immunomodulatory Peptide LL-37 in Human Monocytes and Formation of LL-37 Positive Large Vesicles in Differentiated Monoosteophils. Bone Rep..

[35] Jana J., Kar R.K., Ghosh A., Biswas A., Ghosh S., Bhunia A., Chatterjee S. (2013). Human Cathelicidin Peptide LL37 Binds Telomeric G-Quadruplex. Mol. BioSyst..

[36] Muñoz M., Craske M., Severino P., De Lima T.M., Labhart P., Chammas R., Velasco I.T., Machado M.C.C., Egan B., Nakaya H.I. (2016). Antimicrobial Peptide LL-37 Participates in the Transcriptional Regulation of Melanoma Cells. J. Cancer.

[37] Bertheloot D., Latz E., Franklin B.S. (2021). Necroptosis, Pyroptosis and Apoptosis: An Intricate Game of Cell Death. Cell. Mol. Immunol..

[38] Czabotar P.E., Garcia-Saez A.J. (2023). Mechanisms of BCL-2 Family Proteins in Mitochondrial Apoptosis. Nat. Rev. Mol. Cell Biol..

[39] Glover H.L., Schreiner A., Dewson G., Tait S.W.G. (2024). Mitochondria and Cell Death. Nat. Cell Biol..

[40] Barlow P.G., Li Y., Wilkinson T.S., Bowdish D.M.E., Lau Y.E., Cosseau C., Haslett C., Simpson A.J., Hancock R.E.W., Davidson D.J. (2006). The Human Cationic Host Defense Peptide LL-37 Mediates Contrasting Effects on Apoptotic Pathways in Different Primary Cells of the Innate Immune System. J. Leukoc. Biol..

[41] Lau Y.E., Bowdish D.M.E., Cosseau C., Hancock R.E.W., Davidson D.J. (2006). Apoptosis of Airway Epithelial Cells. Am. J. Respir. Cell Mol. Biol..

[42] Chen X., Ji S., Si J., Zhang X., Wang X., Guo Y., Zou X. (2020). Human Cathelicidin Antimicrobial Peptide Suppresses Proliferation, Migration and Invasion of Oral Carcinoma HSC-3 Cells via a Novel Mechanism Involving Caspase-3 Mediated Apoptosis. Mol. Med. Rep..

[43] Otera H., Ohsakaya S., Nagaura Z., Ishihara N., Mihara K. (2005). Export of Mitochondrial AIF in Response to Proapoptotic Stimuli Depends on Processing at the Intermembrane Space. EMBO J..

[44] Nguyen T.N.A., Lai H.-T., Fernandes R., Dall’Olio F.G., Blériot C., Ha-Duong T., Brenner C. (2025). Apoptosis-Inducing Factor (AIF) at the Crossroad of Cell Survival and Cell Death: Implications for Cancer and Mitochondrial Diseases. Cell Commun. Signal..

[45] Li L.Y., Luo X., Wang X. (2001). Endonuclease G Is an Apoptotic DNase When Released from Mitochondria. Nature.

[46] Bankell E., Dahl S., Gidlöf O., Svensson D., Nilsson B.-O. (2021). LL-37-Induced Caspase-Independent Apoptosis Is Associated with Plasma Membrane Permeabilization in Human Osteoblast-like Cells. Peptides.

[47] Jiang X., Yang C., Qiu J., Ma D., Xu C., Hu S., Han W., Yuan B., Lu Y. (2022). Nanomolar LL-37 Induces Permeability of a Biomimetic Mitochondrial Membrane. Nanoscale.

[48] Ren S.X., Cheng A.S.L., To K.F., Tong J.H.M., Li M.S., Shen J., Wong C.C.M., Zhang L., Chan R.L.Y., Wang X.J. (2012). Host Immune Defense Peptide LL-37 Activates Caspase-Independent Apoptosis and Suppresses Colon Cancer. Cancer Res..

[49] Piktel E., Niemirowicz K., Wnorowska U., Wątek M., Wollny T., Głuszek K., Góźdź S., Levental I., Bucki R. (2016). The Role of Cathelicidin LL-37 in Cancer Development. Arch. Immunol. Ther. Exp..

[50] Lu F., Zhu Y., Zhang G., Liu Z. (2022). Renovation as Innovation: Repurposing Human Antibacterial Peptide LL-37 for Cancer Therapy. Front. Pharmacol..

[51] Bankell E., Liu X., Lundqvist M., Svensson D., Swärd K., Sparr E., Nilsson B.-O. (2022). The Antimicrobial Peptide LL-37 Triggers Release of Apoptosis-Inducing Factor and Shows Direct Effects on Mitochondria. Biochem. Biophys. Rep..

[52] Roufayel R., Younes K., Al-Sabi A., Murshid N. (2022). BH3-Only Proteins Noxa and Puma Are Key Regulators of Induced Apoptosis. Life.

[53] Habibi A., Davari A., Isazadeh K. (2024). A Novel LL-37@NH2@Fe3O4 Inhibits the Proliferation of the Leukemia K562 Cells: In-Vitro Study. Sci. Rep..

[54] Ding X., Bian D., Li W., Xie Y., Li X., Lv J., Tang R. (2021). Host Defense Peptide LL-37 Is Involved in the Regulation of Cell Proliferation and Production of pro-Inflammatory Cytokines in Hepatocellular Carcinoma Cells. Amino Acids.

[55] Lee W.Y., Savage J.R., Zhang J., Jia W., Oottamasathien S., Prestwich G.D. (2013). Prevention of Anti-Microbial Peptide LL-37-Induced Apoptosis and ATP Release in the Urinary Bladder by a Modified Glycosaminoglycan. PLoS ONE.

[56] Zuo Y., Dang R., Peng H., Hu P., Yang Y. (2024). LL37-mtDNA Regulates Viability, Apoptosis, Inflammation, and Autophagy in Lipopolysaccharide-Treated RLE-6TN Cells by Targeting Hsp90aa1. Open Life Sci..

[57] Pinheiro Da Silva F., Medeiros M.C.R., Santos Â.B.G.D., Ferreira M.A., Garippo A.L., Chammas R., Caldini E., Velasco I.T., Possolo De Souza H., Machado M.C.C. (2013). Neutrophils LL-37 Migrate to the Nucleus during Overwhelming Infection. Tissue Cell.

[58] Kaiserman D., Bird P.I. (2010). Control of Granzymes by Serpins. Cell Death Differ..

[59] Okumura K., Itoh A., Isogai E., Hirose K., Hosokawa Y., Abiko Y., Shibata T., Hirata M., Isogai H. (2004). C-Terminal Domain of Human CAP18 Antimicrobial Peptide Induces Apoptosis in Oral Squamous Cell Carcinoma SAS-H1 Cells. Cancer Lett..

[60] Kuroda K., Fukuda T., Yoneyama H., Katayama M., Isogai H., Okumura K., Isogai E. (2012). Anti-Proliferative Effect of an Analogue of the LL-37 Peptide in the Colon Cancer Derived Cell Line HCT116 P53+/+ and P53−/−. Oncol. Rep..

[61] Kuroda K., Okumura K., Isogai H., Isogai E. (2015). The Human Cathelicidin Antimicrobial Peptide LL-37 and Mimics Are Potential Anticancer Drugs. Front. Oncol..

[62] Ren S.X., Shen J., Cheng A.S.L., Lu L., Chan R.L.Y., Li Z.J., Wang X.J., Wong C.C.M., Zhang L., Ng S.S.M. (2013). FK-16 Derived from the Anticancer Peptide LL-37 Induces Caspase-Independent Apoptosis and Autophagic Cell Death in Colon Cancer Cells. PLoS ONE.

[63] Yang T., Li J., Jia Q., Zhan S., Zhang Q., Wang Y., Wang X. (2021). Antimicrobial Peptide 17BIPHE2 Inhibits the Proliferation of Lung Cancer Cells in Vitro and in Vivo by Regulating the ERK Signaling Pathway. Oncol. Lett..

[64] Sengupta P., Banerjee N., Roychowdhury T., Dutta A., Chattopadhyay S., Chatterjee S. (2018). Site-Specific Amino Acid Substitution in Dodecameric Peptides Determines the Stability and Unfolding of *c-MYC* Quadruplex Promoting Apoptosis in Cancer Cells. Nucleic Acids Res..

[65] Açil Y., Torz K., Gülses A., Wieker H., Gerle M., Purcz N., Will O.M., Eduard Meyer J., Wiltfang J. (2018). An Experimental Study on Antitumoral Effects of KI-21-3, a Synthetic Fragment of Antimicrobial Peptide LL-37, on Oral Squamous Cell Carcinoma. J. Cranio-Maxillofac. Surg..

[66] Xu C.-Y., Li Z.-J., Hu W.-Z. (2017). Up-Regulation of HCRP1 Inhibits Proliferation and Invasion in Glioma Cells via Suppressing the ERK and AKT Signaling Pathways. Biomed. Pharmacother..

[67] McCubrey J.A., Steelman L.S., Chappell W.H., Abrams S.L., Wong E.W.T., Chang F., Lehmann B., Terrian D.M., Milella M., Tafuri A. (2007). Roles of the Raf/MEK/ERK Pathway in Cell Growth, Malignant Transformation and Drug Resistance. Biochim. Biophys. Acta Mol. Cell Res..

[68] Weber G., Chamorro C.I., Granath F., Liljegren A., Zreika S., Saidak Z., Sandstedt B., Rotstein S., Mentaverri R., Sánchez F. (2009). Human Antimicrobial Protein hCAP18/LL-37 Promotes a Metastatic Phenotype in Breast Cancer. Breast Cancer Res..

[69] Heilborn J.D., Nilsson M.F., Jimenez C.I.C., Sandstedt B., Borregaard N., Tham E., Sørensen O.E., Weber G., Ståhle M. (2005). Antimicrobial Protein hCAP18/LL-37 Is Highly Expressed in Breast Cancer and Is a Putative Growth Factor for Epithelial Cells. Int. J. Cancer.

[70] Nagaoka I., Tamura H., Hirata M. (2006). An Antimicrobial Cathelicidin Peptide, Human CAP18/LL-37, Suppresses Neutrophil Apoptosis via the Activation of Formyl-Peptide Receptor-Like 1 and P2X7. J. Immunol..

[71] Cattaneo F., Parisi M., Ammendola R. (2013). Distinct Signaling Cascades Elicited by Different Formyl Peptide Receptor 2 (FPR2) Agonists. Int. J. Mol. Sci..

[72] Cancilla D., Rettig M.P., DiPersio J.F. (2020). Targeting CXCR4 in AML and ALL. Front. Oncol..

[73] Podaza E., Palacios F., Croci D.O., Risnik D., Yan X.J., Almejún M.B., Colado A., Elías E.E., Borge M., Morande P.E. (2020). Expression and Function of Cathelicidin hCAP18/LL-37 in Chronic Lymphocytic Leukemia. Haematologica.

[74] Akgul C., Moulding D.A., Edwards S.W. (2001). Molecular Control of Neutrophil Apoptosis. FEBS Lett..

[75] Nolan B., Duffy A., Paquin L., De M., Collette H., Graziano C.M., Bankey P. (1999). Mitogen-Activated Protein Kinases Signal Inhibition of Apoptosis in Lipopolysaccharide-Stimulated Neutrophils. Surgery.

[76] Nagaoka I., Suzuki K., Niyonsaba F., Tamura H., Hirata M. (2012). Modulation of Neutrophil Apoptosis by Antimicrobial Peptides. Int. Sch. Res. Not..

[77] Yang Y., Huang C., Hui L., Song Y., Fu Y., Li M., Yang H., Wu J., Sun J., Xu W. (2023). Cathelicidins Target HSP60 To Restrict CVB3 Transmission via Disrupting the Exosome and Reducing Cardiomyocyte Apoptosis. J. Virol..

[78] Suzuki K., Murakami T., Kuwahara-Arai K., Tamura H., Hiramatsu K., Nagaoka I. (2011). Human Anti-Microbial Cathelicidin Peptide LL-37 Suppresses the LPS-Induced Apoptosis of Endothelial Cells. Int. Immunol..

[79] Suzuki K., Ohkuma M., Nagaoka I. (2025). Human Cathelicidin Peptide LL-37 Induces Endothelial-to-Mesenchymal Transition. Biosci. Biotechnol. Biochem..

[80] Hu Z., Murakami T., Suzuki K., Tamura H., Kuwahara-Arai K., Iba T., Nagaoka I. (2014). Antimicrobial Cathelicidin Peptide LL-37 Inhibits the LPS/ATP-Induced Pyroptosis of Macrophages by Dual Mechanism. PLoS ONE.

[81] Hu Z., Murakami T., Suzuki K., Tamura H., Reich J., Kuwahara-Arai K., Iba T., Nagaoka I. (2016). Antimicrobial Cathelicidin Peptide LL-37 Inhibits the Pyroptosis of Macrophages and Improves the Survival of Polybacterial Septic Mice. Int. Immunol..

[82] Wang Q., Wen W., Zhou L., Liu F., Ren X., Yu L., Chen H., Jiang Z. (2024). LL-37 Improves Sepsis-Induced Acute Lung Injury by Suppressing Pyroptosis in Alveolar Epithelial Cells. Int. Immunopharmacol..

[83] Thomas A.J., Pulsipher A., Davis B.M., Alt J.A. (2017). LL-37 Causes Cell Death of Human Nasal Epithelial Cells, Which Is Inhibited with a Synthetic Glycosaminoglycan. PLoS ONE.

[84] Suzuki K., Ohkuma M., Someya A., Mita T., Nagaoka I. (2022). Human Cathelicidin Peptide LL-37 Induces Cell Death in Autophagy-Dysfunctional Endothelial Cells. J. Immunol..

[85] Xi L., Du J., Xue W., Shao K., Jiang X., Peng W., Li W., Huang S. (2024). Cathelicidin LL-37 Promotes Wound Healing in Diabetic Mice by Regulating TFEB-Dependent Autophagy. Peptides.

[86] Rekha R.S., Padhi A., Frengen N., Hauenstein J., Végvári Á., Agerberth B., Månsson R., Guðmundsson G.H., Bergman P. (2025). The Di-Leucine Motif in the Host Defense Peptide LL-37 Is Essential for Initiation of Autophagy in Human Macrophages. Cell Rep..

[87] Zhang Z., Cherryholmes G., Shively J.E. (2008). Neutrophil Secondary Necrosis Is Induced by LL-37 Derived from Cathelicidin. J. Leukoc. Biol..

[88] Li H.-N., Barlow P.G., Bylund J., Mackellar A., Björstad Å., Conlon J., Hiemstra P.S., Haslett C., Gray M., Simpson A.J. (2009). Secondary Necrosis of Apoptotic Neutrophils Induced by the Human Cathelicidin LL-37 Is Not Proinflammatory to Phagocytosing Macrophages. J. Leukoc. Biol..

[89] Svensson D., Nilsson B.-O. (2025). Human Antimicrobial/Host Defense Peptide LL-37 May Prevent the Spread of a Local Infection through Multiple Mechanisms: An Update. Inflamm. Res..

[90] Zhang J., Hou Y., Yasuda T., Chow S.Y.A., Ito S., Otsuka M., Ikeuchi Y., Li X., Sugihara K. (2026). Heteroaggregation of Antimicrobial Peptides LL-37 and HNP-1 Drives Cooperative Neutralization of Cytotoxicity. JACS Au.

[91] Chieosilapatham P., Ikeda S., Ogawa H., Niyonsaba F. (2018). Tissue-Specific Regulation of Innate Immune Responses by Human Cathelicidin LL-37. Curr. Pharm. Des..

[92] Zhang Q., Choi K., Wang X., Xi L., Lu S. (2025). The Contribution of Human Antimicrobial Peptides to Fungi. Int. J. Mol. Sci..

[93] Wu W.K.K., Sung J.J.Y., To K.F., Yu L., Li H.T., Li Z.J., Chu K.M., Yu J., Cho C.H. (2010). The Host Defense Peptide LL-37 Activates the Tumor-suppressing Bone Morphogenetic Protein Signaling via Inhibition of Proteasome in Gastric Cancer Cells. J. Cell. Physiol..

[94] Ou H., Zhu H., Zhu W., He B., Wu S. (2021). Cathelicidin LL-37 Induces Apoptosis of Gastric Cancer AGS Cells by Activating P53 Signaling Pathway. Acta Univ. Med. Anhui.

[95] Kuroda K., Fukuda T., Krstic-Demonacos M., Demonacos C., Okumura K., Isogai H., Hayashi M., Saito K., Isogai E. (2017). MiR-663a Regulates Growth of Colon Cancer Cells, after Administration of Antimicrobial Peptides, by Targeting CXCR4-P21 Pathway. BMC Cancer.

[96] Lu F., Liu Z., Jiang H., Zheng H., Su W., Zhou B. (2026). LL-37 Inhibits Osteosarcoma Progression by Suppressing SQLE-Mediated Cholesterol Synthesis via the PTEN/AKT/mTOR Pathway. J. Bone Oncol..

[97] Sun Q., Peng G., Wu Z., Cui L., Zhao W., Abudouwanli A., Yang M., Wang S., Tan Y., Ogawa H. (2026). Melanoma Exosomal Hsa-miR-221-5p Suppresses Keratinocyte LL-37 to Promote Tumor Progression. Cell Rep..

[98] Jia J., Zheng Y., Wang W., Shao Y., Li Z., Wang Q., Wang Y., Yan H. (2017). Antimicrobial Peptide LL-37 Promotes YB-1 Expression, and the Viability, Migration and Invasion of Malignant Melanoma Cells. Mol. Med. Rep..

[99] Zhang Z., Chen W.-Q., Zhang S.-Q., Bai J.-X., Lau C.-L., Sze S.C.-W., Yung K.K.-L., Ko J.K.-S. (2022). The Human Cathelicidin Peptide LL-37 Inhibits Pancreatic Cancer Growth by Suppressing Autophagy and Reprogramming of the Tumor Immune Microenvironment. Front. Pharmacol..

[100] Sainz B., Alcala S., Garcia E., Sanchez-Ripoll Y., Azevedo M.M., Cioffi M., Tatari M., Miranda-Lorenzo I., Hidalgo M., Gomez-Lopez G. (2015). Microenvironmental hCAP-18/LL-37 Promotes Pancreatic Ductal Adenocarcinoma by Activating Its Cancer Stem Cell Compartment. Gut.

[101] Zhang H., Yuan X., Yang Y., Wanyan Y., Tao L., Chen Y. (2023). Cathelicidin LL-37 Promotes EMT, Migration and Metastasis of Hepatocellular Carcinoma Cells in Vitro and Mouse Model. Cell Adhes. Migr..

[102] Cha H.-R., Lee J.H., Hensel J.A., Sawant A.B., Davis B.H., Lee C.M., Deshane J.S., Ponnazhagan S. (2016). Prostate Cancer-Derived Cathelicidin-Related Antimicrobial Peptide Facilitates Macrophage Differentiation and Polarization of Immature Myeloid Progenitors to Protumorigenic Macrophages: The Role of CRAMP in Prostate Cancer. Prostate.

[103] Hensel J.A., Chanda D., Kumar S., Sawant A., Grizzle W.E., Siegal G.P., Ponnazhagan S. (2011). LL-37 as a Therapeutic Target for Late Stage Prostate Cancer. Prostate.

[104] Wollny T., Wnorowska U., Piktel E., Suprewicz Ł., Król G., Głuszek K., Góźdź S., Kopczyński J., Bucki R. (2022). Sphingosine-1-Phosphate-Triggered Expression of Cathelicidin LL-37 Promotes the Growth of Human Bladder Cancer Cells. Int. J. Mol. Sci..

[105] Pan W.L., Wang Y., Hao Y., Wong J.H., Chan W.C., Wan D.C.-C., Ng T.B. (2018). Overexpression of CXCR4 Synergizes with LL-37 in the Metastasis of Breast Cancer Cells. Biochim. Biophys. Acta Mol. Basis Dis..

[106] Gambade A., Zreika S., Guéguinou M., Chourpa I., Fromont G., Bouchet A.M., Burlaud-Gaillard J., Potier-Cartereau M., Roger S., Aucagne V. (2016). Activation of TRPV2 and BKCa Channels by the LL-37 Enantiomers Stimulates Calcium Entry and Migration of Cancer Cells. Oncotarget.

[107] Ji P., Zhou Y., Yang Y., Wu J., Zhou H., Quan W., Sun J., Yao Y., Shang A., Gu C. (2019). Myeloid Cell-Derived LL-37 Promotes Lung Cancer Growth by Activating Wnt/β-Catenin Signaling. Theranostics.

[108] Von Haussen J., Koczulla R., Shaykhiev R., Herr C., Pinkenburg O., Reimer D., Wiewrodt R., Biesterfeld S., Aigner A., Czubayko F. (2008). The Host Defence Peptide LL-37/hCAP-18 Is a Growth Factor for Lung Cancer Cells. Lung Cancer.

[109] Coffelt S.B., Waterman R.S., Florez L., Bentrup K.H.Z., Zwezdaryk K.J., Tomchuck S.L., LaMarca H.L., Danka E.S., Morris C.A., Scandurro A.B. (2008). Ovarian Cancers Overexpress the Antimicrobial Protein hCAP-18 and Its Derivative LL-37 Increases Ovarian Cancer Cell Proliferation and Invasion. Int. J. Cancer.

[110] Wang G., Li Y., Yang G., Yang T., He L., Wang Y. (2021). Cathelicidin Antimicrobial Peptide (CAMP) Gene Promoter Methylation Induces Chondrocyte Apoptosis. Hum. Genom..

[111] Porter R.J., Murray G.I., Alnabulsi A., Humphries M.P., James J.A., Salto-Tellez M., Craig S.G., Wang J.M., Yoshimura T., McLean M.H. (2021). Colonic Epithelial Cathelicidin (LL-37) Expression Intensity Is Associated with Progression of Colorectal Cancer and Presence of CD8^+^ T Cell Infiltrate. J. Pathol. Clin. Res..

[112] Dho S.H., Cho M., Woo W., Jeong S., Kim L.K. (2025). Caspases as Master Regulators of Programmed Cell Death: Apoptosis, Pyroptosis and Beyond. Exp. Mol. Med..

[113] Thome M., Schneider P., Hofmann K., Fickenscher H., Meinl E., Neipel F., Mattmann C., Burns K., Bodmer J.-L., Schröter M. (1997). Viral FLICE-Inhibitory Proteins (FLIPs) Prevent Apoptosis Induced by Death Receptors. Nature.

[114] Hüttmann J., Krause E., Schommartz T., Brune W. (2016). Functional Comparison of Molluscum Contagiosum Virus vFLIP MC159 with Murine Cytomegalovirus M36/vICA and M45/vIRA Proteins. J. Virol..

[115] Arnoult D., Bartle L.M., Skaletskaya A., Poncet D., Zamzami N., Park P.U., Sharpe J., Youle R.J., Goldmacher V.S. (2004). Cytomegalovirus Cell Death Suppressor vMIA Blocks Bax- but Not Bak-Mediated Apoptosis by Binding and Sequestering Bax at Mitochondria. Proc. Natl. Acad. Sci. USA.

[116] Niemirowicz K., Prokop I., Wilczewska A., Wnorowska U., Piktel E., Wątek M., Savage P., Bucki R. (2015). Magnetic Nanoparticles Enhance the Anticancer Activity of Cathelicidin LL-37 Peptide against Colon Cancer Cells. Int. J. Nanomed..

